# Pituitary Adenoma and the Chemokine Network: A Systemic View

**DOI:** 10.3389/fendo.2015.00141

**Published:** 2015-09-11

**Authors:** Fabio Grizzi, Elena Monica Borroni, Alessandro Vacchini, Dorina Qehajaj, Manuela Liguori, Sanja Stifter, Maurizio Chiriva-Internati, Antonio Di Ieva

**Affiliations:** ^1^Department of Inflammation and Immunology, Humanitas Clinical and Research Center, Milan, Italy; ^2^Department of Medical Biotechnologies and Translational Medicine, University of Milan, Milan, Italy; ^3^Department of Pathology, University of Rijeka, Rijeka, Croatia; ^4^Division of Hematology and Oncology, Texas Tech University Health Sciences Center, Lubbock, TX, USA; ^5^Department of Neurosurgery, Australian School of Advanced Medicine, Macquarie University Hospital, Sydney, NSW, Australia; ^6^Garvan Institute of Medical Research, Sydney, NSW, Australia

**Keywords:** pituitary gland, adenoma, chemokines, systems biology, hormones

Pituitary adenomas (PAs) are adenohypophysial neoplasms, representing ~10–15% of all intracranial tumors, which are found to occur in almost 20% of the general population (1, 2). Although recognized as benign lesions, 20–45% of PAs are invasive and some exhibit clinically aggressive behavior (1, 3). Advancements have allowed the pathological classification of PAs from a histochemical classification (i.e., acidophilic, basophilic, and chromophobic PAs) to an immunohistochemical-based one, which essentially recognizes PAs as lactotrophic, somatotrophic, corticotrophic, gonadotrophic, thyrotrophic, and null cell adenomas (4). Electron microscopy has identified additional subtypes, based on the appearance of specific morphology/arrangement of ubiquitous cytoplasmic constituents (5). It is now accepted that the hypothalamic–anterior pituitary axis integrates a set of stimulatory and inhibitory central and peripheral signals to synthesize and secrete hormones by highly differentiated cell types, namely somatotrophs, gonadotrophs, lactotrophs, thyrotrophs, and corticotrophs. Each of these cell types expresses unique G protein-coupled receptors (GPCRs), which are specific for hypothalamic releasing and inhibiting hormones. These peptides traverse the vascular system that connects the hypothalamus with the anterior pituitary gland and impinge upon the pituitary cells to regulate the synthesis and secretion of anterior pituitary hormones (6). It has been demonstrated that PAs arise from any of these cells as monoclonal neoplasms (7). The functional classification of PAs has also been facilitated by the measurement of circulating trophic and target hormone concentrations (6). Several hallmarks of PAs (i.e., benign nature, slow growth) point to a unique growth behavior distinct from that of other endocrine and non-endocrine malignancies (8). The general failure to proceed to true malignancy with demonstrable extra-cranial metastases remains an intriguing feature not completely investigated yet (8). Atypical and/or aggressive PAs can share some histological features with carcinomas, including atypical morphologic appearances, elevated mitotic index (i.e., Ki-67 labeling index >3%), or extensive nuclear staining for p53 (9). Nonetheless, the endocrine signs due to hormonal hypersecretion or pituitary deficit, pituitary abnormal growth, or invasion represent a greater clinical and therapeutic challenge (8). The subset of PAs with clinically aggressive behavior should be promptly identified, as patients with such tumors might require closer clinical, biochemical, and imaging surveillance or multimodal therapeutic treatments. Despite the paramount importance of identifying specific criteria for tumor aggressiveness, no clinical signs, biochemical biomarkers, or imaging techniques have been universally accepted and no reliable histological biomarker or classification system for characterizing pituitary aggressive neoplasms are available today (10). It has also been suggested that the histological features of “typical” and “atypical” PAs as defined by the World Health Organization (WHO) do not entirely correlate with clinical outcome. In particular, some typical PAs have an aggressive behavior while many atypical PAs lack an aggressive clinical dynamics (1, 7). Additionally, the lack of standardization of the terminology in the literature remains a source of confusion (i.e., the terms “aggressive” and “invasive” are often used synonymously).

Although the molecular pathogenesis of PAs is still unclear, it is now accepted that PAs onset is related to proto-oncogene mutations, overexpression of activating genes, or loss of tumor suppressor genes (11). The “initiating” events cause a proliferative “gain of function” in single pituitary cells, subsequently induced to clonal expansion by tumor-promoting molecules. Recently, several studies have shown the pivotal role of “epigenetic modifications” (11, 12), microRNA (miRNAs), and long non-coding RNAs (lncRNAs) in the pathogenesis of PAs (13–17). Other promoting factors, including hypothalamic hormones, locally produced growth factors (i.e., EGF, βFGF, NGF, and TGF), cytokines [interleukin (IL)-1, IL-2, and IL-6], and chemokines have been shown to influence pituitary tumor progression (18, 19).

Chemokines represent a category of inflammatory mediators with a prominent role in leukocyte migration and angiogenesis, exerted through the activation of dedicated seven-transmembrane domain receptors, known as GPCRs (20, 21). Beyond conventional chemokine receptors, which directly induce cell migration through Gi-mediated signaling events, a set of atypical chemokine receptors (ACKRs) with no Gi-mediated signaling activity and devoid of chemotactic activity have been described (22). Recent data indicate that ACKRs fulfill their biological functions by contributing to generate and maintain functional chemokine patterns in tissues by means of different biochemical properties, including removal, transport, or concentration of their cognate ligands (23). Deregulated expression of chemokines and their receptors is involved in the development of many human diseases, including autoimmune and chronic inflammatory diseases, immunodeficiency, and cancer (24). In the adult central nervous system (CNS), chemokines and their receptors are involved in developmental, physiological processes and nervous system disorders, including neuroinflammatory and neurodegenerative diseases, HIV-associated neuropathology, and brain tumors (25–27). In particular, the C–X–C chemokine receptor type 4 (CXCR4) and its ligand stromal cell-derived factor 1 [SDF1, also known as C–X–C motif chemokine 12 (CXCL12)] are expressed by neuronal, astroglial, and microglial cells in the adult brain and play a critical role in supporting cell growth and survival, and directing cell migration during embryonic brain development (28–31). Moreover, the CXCL12/CXCR4 axis has been found in malignant tumors, including meningiomas, gliomas, where it crucially affects tumor progression by controlling cancer cell survival, proliferation, and migration, and, indirectly, regulating angiogenesis and vasculogenesis or recruiting fibroblasts, endothelial, mesenchymal, and immune cells in tumor microenvironment (32, 33). Regulation of CXCL12 is unique in that it may control its own expression levels and fine tune its biological functions by means of its binding to the ACKR CXCR7/ACKR3 in addition to CXCR4, which has been reported to regulate angiogenesis in different human tumors through its own signaling activity (34). ACKR3 has been found simultaneously expressed with CXCL12 and CXCR4 in a cohort of brain metastases (35). Therefore, the CXCL12/CXCR4/ACKR3 pathway could be investigated to target tumor growth, invasion, and proliferation of metastatic cells. Additionally, it opens new perspectives in the development of specific therapeutic approaches that include chemokine-based drugs. Interestingly, the CXCR4 antagonist Plerixafor (also known as AMD3100) is already Food and Drugs Administration-approved for stem cell mobilization in several tumors, including non-Hodgkin’s lymphoma and multiple myeloma (36, 37) and its systemic administration has been reported to inhibit growth of intracranial glioblastoma and medulloblastoma xenografts by increasing apoptosis and decreasing the proliferation of tumor cells (38).

Recently, several chemokines (i.e., CXCL1, CXCL10, CXCL12) have been identified as novel regulators of the hypothalamic–hypophysial axis. However, only a few studies addressed their role in the regulation of normal and tumor pituitary cell functions. In particular,

(a) CXCL1 is indeed expressed in the posterior pituitary gland, in the paraventricular nucleus of the hypothalamus and the median eminence. In response to stressful stimuli, CXCL1 is released in the median eminence to reach its receptor CXCR2 expressed in pituitary cells and induce the release of PRL and GH along with the inhibition of LH and FSH secretion (39). CXCL1 was detected in a small percentage of human PAs and CXCR2 was identified in both human PAs without a tumor-specific phenotype and natural pituitary tissue (40).
(b) The CXCL10 receptor CXCR3 has been reported to be expressed in corticotrophs, suggesting a possible autocrine/paracrine effect of CXCL10, released from FSH cells, on ACTH-producing cells (41).
(c) Differently from rats (42), CXCR4 expression was confined to a subset of pituitary cells but not to a specific cell sub-population (i.e., GH-, PRL- or ACTH-secreting cells). The CXCR4 ligand CXCL12 has been identified mostly but not exclusively in ACTH-expressing pituitary cells (43, 44). It may be hypothesized that CXCR4/CXCL12 axis could be expressed in undifferentiated and/or progenitor cells and contribute to the paracrine regulation of pituitary hormone secretion. In this perspective, the possibility that pituitary CXCR4 should also be activated by the chemokine derived from the systemic circulation or from hypothalamic terminals cannot be excluded. Recently, several studies have revealed the expression of CXCL12 and/or CXCR4 in human pituitary tumors, suggesting that this chemokine may act as a promoting factor for adenoma development (43). Barbieri et al. have investigated the expression of both CXCL12 and CXCR4 in a cohort of 65 human PAs and 4 normal hypophyses and the potential autocrine–paracrine role of CXCL12 in pituitary cell proliferation (44). They provided the first evidence of CXCL12 and CXCR4 expression in normal and adenomatous human pituitary, and showed that overexpression of both ligand and receptor occurs in all the adenomatous cells compared with normal pituitary cells, thus suggesting their role in providing a gain-of-function to pituitary cells by affecting both cell proliferation and hormone secretion, thus contributing to PA development and/or progression. Differently from normal pituitary cells that do not co-express CXCL12 and CXCR4, concomitant expression of both ligand and receptor occurs in PAs, suggesting that autocrine stimulation of CXCR4 seems to represent a key characteristic of PAs. Xing et al. investigated the expression of CXCR4 and CXCL12 in 35 PAs (21 invasive and 14 non-invasive) patients who underwent surgical resection, and demonstrated that that there was a positive correlation between the level of CXCR4 and CXCL12 and the progression stage of PAs, suggesting that CXCR4 and CXCL12 played a role in the regulation of PAs invasiveness (45). Further studies will be required to verify whether CXCL12 overexpression may cause PAs development or only provide a selective proliferation advantage favoring clonal expansion of cells in which tumor-promoting mechanisms are already activated. Moreover, the general lack of progression from PA to carcinoma and metastatic status suggests further studies to investigate the potential involvement of CXCL12/CXCR4 axis. Interestingly, ACKR3 was expressed by all the PAs, especially in GH and PRL secreting adenomas and it was observed that its expression was significantly higher in macro- than micro-adenomas (46).

It is known that that the concomitant expression of ligand–receptor in the same tumor cells is one of the leading causes of clinical aggressive behavior in various cancer types. It is known that Raf/MEK/ERK and PI3K/Akt pathway deregulation is a common alteration responsible of tumor initiation and progression in melanoma (47). While the pathways are classically activated by growth factors, crosstalk and transactivation mechanisms with neuropeptide–cytokine–chemokines/GPCRs have been increasingly recognized. It has been shown that the overexpression or constitutive activation of receptors for growth factors, cytokines, and chemokines potentiates the activation of Ras/Raf/MEK/ERK pathway also in PA (48–50). Chemokines, particularly CXCL12 signaling via CXCR4 and ACKR3, represent candidate mediators of the above-described intracellular pathways, determining proliferative, antiapoptotic, and angiogenic signals, thus possibly concurring to pituitary tumor development and aggressiveness. Therefore, the better characterization of CXCR4/ACKR3/CXCL12 axis in PAs (Figure 1) could pave the way for novel pharmacological approaches, especially for those adenoma subtypes (i.e., TSH and ACTH-secreting tumors, as well as NFPA) still waiting for efficacious therapeutic drugs. Other studies, however, highlighted that, when considering the complexity of regulatory pathways involved in pituitary cell survival and proliferation, it should be taken into account not only apoptosis but also the cellular “senescence.” Senescence is gaining biological significance also in PAs, whose typical benign nature could result from protective anti-proliferative mechanisms. PAs may be prone to activate senescence-associated pathways, maintaining their benign behavior and preventing malignant transformation. Furthermore, autophagy-related mechanisms in pituitary tumors are still under investigation (51, 52).

**Figure 1:**
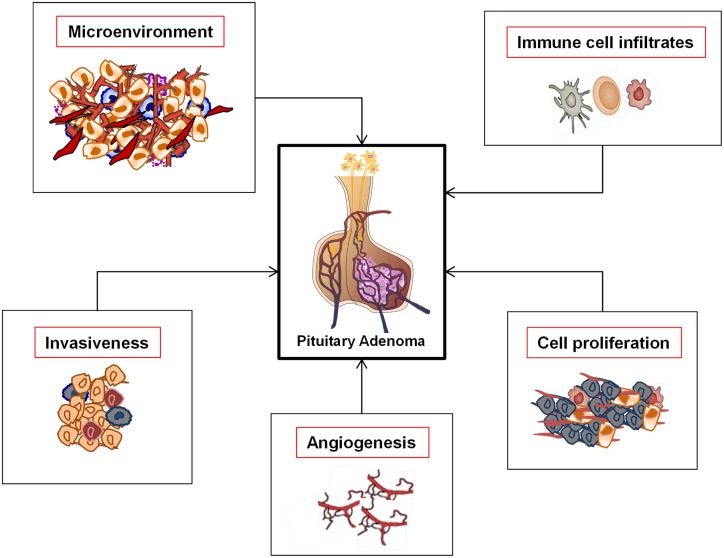
**Functional roles of the CXCR4/ACKR3/CXCL12 axis in pituitary adenomas**.

Cancer research has undergone radical changes over the last few years. The issue today is no longer the amount of molecular, cellular, and clinical information available, but the network interactions among the single components (53, 54). Systems biology is the latest in a series of strategies driven by technological advances that have provided us with a suite of “omics” (55). The “analytic” and the “systemic” approaches are more complementary than opposed, yet neither one is reducible to the other. The analytic approach seeks to reduce a system to its elementary elements in order to study in detail and understand the types of interaction that exist between them. The “additive” laws of elementary properties do not apply in complex systems, i.e., PAs, composed of a large diversity of elements linked together by complex interactions. These systems must be approached by new methods such as those, which the systemic approach groups together. The purpose of the new methods is to consider a system as a “whole,” its complexity, and its own dynamics. In conclusion, although the role of chemokines in PA development has been poorly investigated, the evaluation of CXCR4 and CXCL12 expression in invasive and non-invasive PAs has demonstrated that the percentage of CXCR4- and CXCL12 positive cells was significantly higher in invasive PAs. Therefore, the correlation of CXCR4 and CXCL12 expression levels and tumor invasiveness might be proposed as potential early diagnostic biomarkers (56). Scientific advances are revealing the complexity of pituitary development and its microenvironment, which is controlled by multiple transcription factors and signaling molecules. These results strengthen the idea that to target chemokine networks might represent a novel therapeutic approach for PAs.

## Conflict of Interest Statement

The authors declare that the research was conducted in the absence of any commercial or financial relationships that could be construed as a potential conflict of interest.
